# Juvenile/adult-type galactosialidosis with a homozygous *CTSA* variant without consanguinity

**DOI:** 10.1038/s41439-025-00324-0

**Published:** 2025-09-26

**Authors:** Machiko Toki, Kazushige Tsunoda, Tetsumin So, Motomichi Kosuga, Torayuki Okuyama, Masashi Miharu, Tomonobu Hasegawa, Kazuki Yamazawa

**Affiliations:** 1 Department of Pediatrics, NHO Tokyo Medical Center, Tokyo, Japan; 2 Division of Vision Research, National Institute of Sensory Organs, NHO Tokyo Medical Center, Tokyo, Japan; 3 Division of Medical Genetics, National Center for Child Health and Development, Tokyo, Japan; 4 Department of Pediatrics and Clinical Genomics, Saitama Medical University, Saitama, Japan; 5 Department of Pediatrics, Keio University School of Medicine, Tokyo, Japan; 6 Kashiwa Tanaka Hospital, Chiba, Japan; 7 Department of Medical Genetics, NHO Tokyo Medical Center, Tokyo, Japan

**Keywords:** Disease genetics, Consanguinity, Genetics research, Metabolic syndrome

## Abstract

Here we report a Japanese patient with juvenile/adult-type galactosialidosis carrying a homozygous c.692+3A>G *CTSA* variant. Comprehensive genetic analyses including exome sequencing, chromosomal microarray and homozygosity mapping supported biallelic inheritance of this variant and suggested a founder effect in the Japanese population. Clinically, the patient exhibited typical features of the juvenile/adult-type galactosialidosis, with growth impairment noted during adolescence as a less conspicuous but relevant observation.

Galactosialidosis (GS) is a lysosomal accumulation disorder caused by pathogenic variants in the *CTSA* gene located at 20q13.12. *CTSA* encodes the lysosomal protective protein/cathepsin A (PPCA), which has both catalytic and protective functions, acting as an acid carboxypeptidase, neutral serine esterase and deamidase^1^. PPCA also forms a lysosomal multienzyme complex with β-galactosidase (GLB1) and *N*-acetyl alpha neuraminidase 1 (NEU1), playing a critical role in stabilizing GLB1 and activating NEU1^2^. Loss of PPCA due to alterations in the *CTSA* gene results in its own functional defects as well as a secondary GLB1/NEU1 combined deficiency^3^. The combined GLB1/NEU1 deficiency leads to the lysosomal accumulation of undegraded sialylated oligosaccharides, glycoproteins and glycolipids in various tissues, ultimately causing cytotoxicity.

Loss-of-function variants in *CTSA* can cause GS, characterized by a range of symptoms affecting the central nervous system, as well as the vision and hearing, cardiovascular, gastrointestinal, urinary, skin, blood, skeletal and connective tissue systems. It is classified into three phenotypic subtypes—early infantile, late infantile and juvenile/adult—based on the age of onset. The early infantile type is the most severe phenotype, presenting with fetal hydrops, cherry-red spots, visceral enlargement, global developmental delay, coarse facial features and skeletal dysplasia, resulting in early death. The late infantile type has milder symptoms than the early infantile type and is typically characterized by corneal opacities, cardiac involvement and visceromegaly. Additional features may include intellectual disabilities, hearing loss and a cherry-red spot. Symptoms usually appear around 2 years of age. The juvenile/adult-type differs from the other two forms, with onset typically in adolescence and presenting with myoclonus, ataxia, neurological deterioration, skeletal abnormalities and angiokeratoma. Affected individuals often develop a cherry-red spot, vision loss and hearing loss. Although the age of onset varies, the average is around 16 years^4,5^.

According to a recent review^5^, 142 patients with GS had been reported in the literature by 2019, of whom 57 (40.1%) were Japanese. Notably, the majority of juvenile/adult-onset cases occurred in individuals of Japanese origin^6^, suggesting the existence of a population-specific genetic factor contributing to the high prevalence of juvenile/adult-type GS in Japan. In this context, among the causative variants in the *CTSA* gene, c.692+3A>G has been identified most frequently and exclusively in Japanese patients with juvenile/adult-type GS, with 19 reported cases, including 10 homozygotes^3,6–12^. This predominance further supports the notion of a population-specific founder effect contributing to the high prevalence of juvenile/adult-type GS in Japan. However, although this variant is frequently observed, it remains to be elucidated whether the homozygosity in these cases resulted from consanguinity, hemizygosity due to an allelic deletion or uniparental isodisomy. Moreover, phenotypic characterization of these individuals has also been insufficient. Here, we report the 11th Japanese patient with juvenile/adult-type GS carrying a homozygous c.692+3A>G variant in *CTSA*, along with detailed genetic and phenotypic analyses.

A 21-year-old Japanese woman was referred for evaluation due to visual impairment. She was the first-born of dizygotic twins, and her twin brother was asymptomatic. Her parents, who are unrelated, originated from the same prefecture in Japan. Generalized angiokeratomas, particularly on the palms and soles of the feet, appeared in elementary school. At age 16, she developed myoclonus during fine motor attempts. Despite the absence of electroencephalogram abnormalities, antiepileptic drugs (valproic acid and clonazepam) were initiated, under a provisional diagnosis of myoclonus epilepsy. By age 17, while still able to perform daily activities, she began experiencing difficulties with reading and writing. She could not pick up small objects on the first attempt, and by the evening, she often experienced such severe fatigue that walking required frequent pauses. At this age, her first brain magnetic resonance imaging showed no abnormalities. At age 19, she developed night blindness, visual loss and color blindness, all of which progressively worsened. At age 21, ophthalmological examination revealed cherry-red spots on both eyes (Fig. 1A), leading to referral to our department for further diagnostic evaluation. Physical examination showed angiokeratomas on the face, palms and soles (Fig. 1B), with no hepatosplenomegaly or thyroid enlargement. Neurological examination indicated full but slightly clumsy extraocular movements, prompt pupillary reflexes, slight clumsiness in tongue movements and slow speech. Deep tendon reflexes were normal, but there was bilateral clumsiness in the finger-to-nose test. Pronation and supination movements were smooth, although she had mild difficulty with writing. She had normal intellectual development, with no cognitive impairment. Radiographs showed kyphosis of the radius and ulna, as well as scoliosis with an 8° Cobb angle at the eighth thoracic vertebra (Fig. 1C, D). Peripheral blood smear revealed vacuolated lymphocytes (Fig. 1E), with no other abnormalities detected. Abdominal ultrasound and repeat brain magnetic resonance imaging at age 21 were unremarkable. Her height was 151 cm (−1.36 s.d.; target range 152–168 cm), and weight was 45.3 kg (−1.12 s.d.). The growth chart is shown in Fig. 1F.

**Fig. 1:**
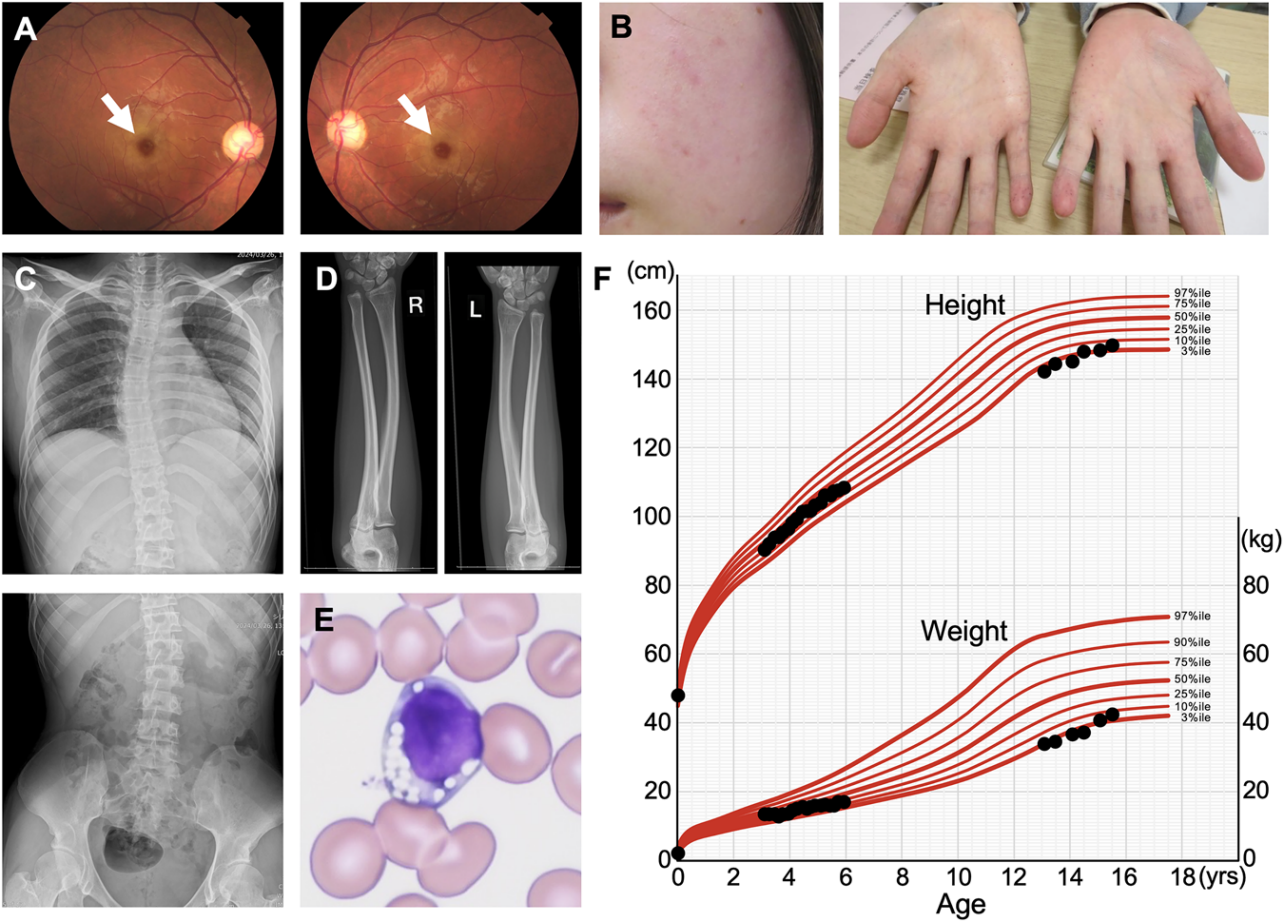
Phenotypic and clinical findings in the patient. **A** Bilateral cherry-red spots observed on funduscopy (arrows). **B** Angiokeratomas on the face (left) and palms (right). **C** Scoliosis involving the thoracic (top) and lumbar (bottom) spine. **D** Kyphosis of the radius and ulna. **E** Peripheral blood smear showing vacuolated lymphocytes. **F** Growth chart demonstrating growth impairment after age 13 years. Note that some data points are missing.

Based on the constellation of her multiple abnormal findings, GS was clinically suspected, and enzyme activity was evaluated. Markedly reduced activities of GLB1 and sialidase in peripheral blood lymphocytes supported the diagnosis of GS (Supplementary Table 1). Subsequently, we proceeded to conduct genetic investigations as described in the Supplementary Methods, after providing genetic counseling and obtaining written consent. Genetic testing for GS identified a splice donor site variant, NG_008291.1(NM_000308.4):c.692+3A>G, in the *CTSA* gene in a homozygous state, thereby confirming the diagnosis of GS. Additional exome sequencing (ES) also detected the same homozygous *CTSA* variant (Fig. 2D), and no other variants were identified that could account for the patient’s phenotype. Results of database searches and in silico analyses of the *CTSA* c.692+3A>G variant are presented in Supplementary Table 2. This variant has been identified exclusively in the Japanese population and has not been reported in other Asian countries or elsewhere worldwide, suggesting a founder effect among Japanese individuals. According to ClinVar and HGMD, c.692+3A>G is classified as pathogenic. Indeed, aberrant splicing resulting from this variant was predicted by SpliceAI^13^ and was experimentally confirmed by Yamazaki et al.^14^. These findings indicate that the residual function of the alternatively spliced transcript may partially compensate for enzyme deficiency and potentially contribute to the milder phenotype observed with this high-frequency variant in Japanese individuals with GS.

**Fig. 2:**
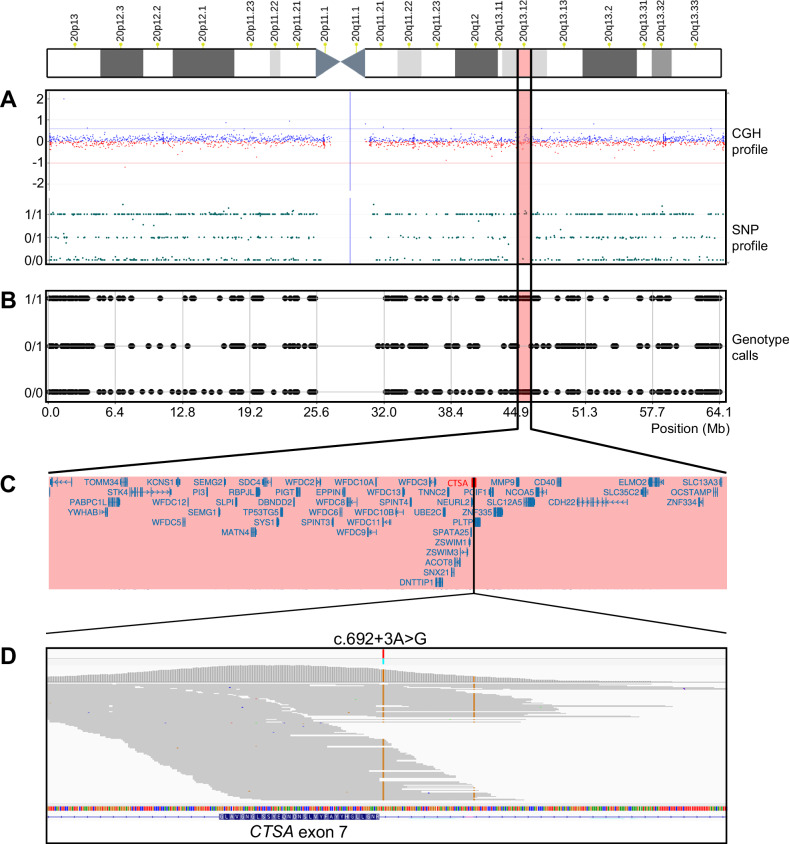
Genetic analysis of the patient. **A** CMA. Top: each blue (positive) and red (negative) dot represents a comparative genomic hybridization (CGH) probe along the length of chromosome 20, with cytobands shown on the *X* axis and the log_2_ ratio on the *Y* axis. Bottom: each green dot represents a single nucleotide polymorphism (SNP) probe, indicating genotypes of homozygotes (0/0 and 1/1) and heterozygotes (0/1) on the *Y* axis. Although no pathogenic copy number variants or ROHs were detected on chromosome 20 under default settings, a region within 20q13.12 lacking heterozygous SNP probes was observed (highlighted in pink). **B** Homozygosity mapping using PLINK. A 1.82-Mb ROH was identified within 20q13.12 (highlighted in pink). Each black dot represents a genotype call obtained from ES, displayed in the same format as in the SNP profile above. **C** Gene content within the ROH. The 1.82-Mb ROH includes 53 RefSeq genes, including *CTSA*. **D** Integrative Genomics Viewer (IGV) view of ES data. A pathogenic variant, c.692+3A>G in *CTSA*, was identified in the homozygous state. Note that a nearby variant, c.692+55A>G, located to the right of c.692+3A>G, is a common benign variant.

Although no parental consanguinity was reported during the clinical interview, both parents originated from the same prefecture, raising the possibility of unrecognized distant relatedness. To investigate this, we additionally performed chromosomal microarray analysis (CMA) and homozygosity mapping using ES data. CMA, performed under default settings, did not detect any pathogenic copy number variants, runs of homozygosity (ROHs) or uniparental isodisomy across the genome, including chromosome 20 where the *CTSA* gene is located (Fig. 2A). By contrast, homozygosity mapping using PLINK^15^ and AutoMap^16^ identified a 1.82-Mb ROH encompassing the *CTSA* gene locus on chromosome 20 (Fig. 2B, C) and a total of 22.07 Mb of ROHs across the autosomes (Supplementary Fig. 1), respectively. ROHs smaller than 3 Mb are commonly observed even in outbred individuals^17^. In such populations, the cumulative length of ROHs is generally less than approximately 50 Mb (refs.^18,19^), suggesting that the homozygous inheritance of the *CTSA* variant in this patient probably occurred by chance rather than as a result of parental consanguinity. Although parental samples were not available for genotyping, it is presumed that the patient is homozygous for the variant due to biallelic inheritance, with one allele transmitted from each parent. Notably, the discrepancy whereby CMA failed to detect ROHs that were identified by PLINK and AutoMap is probably attributable to the lower probe density and limited sensitivity of CMA compared with ES.

Phenotypic features of the patient warrant discussion. In addition to the typical manifestations of GS—such as myoclonus, ataxia, cherry-red spots, angiokeratomas and scoliosis—this patient exhibited growth impairment that became evident by age 13, despite having a normal height during early childhood, as shown in the growth chart (Fig. 1F). Notably, short stature has been documented in 28 of the 142 reported GS cases, including 14 of 74 patients with the juvenile/adult-type^5^. However, it remains unclear whether growth impairment was present but unreported in the remaining cases. Previous reports were published across a wide range of specialty journals—for example, ophthalmology journals emphasizing cherry-red spots, radiology journals focusing on skeletal findings and clinical biochemistry journals reporting enzyme activities—which has resulted in ascertainment bias due to variability in reporting focus. Consequently, growth impairment may not have received sufficient attention so far. From a pediatric perspective, growth impairment may manifest before other clinical features in patients with the juvenile/adult-type and should be recognized as an important aspect of the GS phenotype.

In summary, we report a Japanese patient with juvenile/adult-type GS harboring a homozygous c.692+3A>G *CTSA* variant. Comprehensive genetic analyses, including CMA, ES and homozygosity mapping, supported biallelic inheritance and suggested a founder effect. Growth impairment may represent an early and underrecognized aspect of the phenotype.

## HGV Database

The relevant data from this Data Report are hosted at the Human Genome Variation Database at 10.6084/m9.figshare.hgv.3542.

## Supplementary information

Supplementary Information
