# Supplementary material for: Juvenile/adult-type galactosialidosis with a homozygous CTSA variant without consanguinity
Source: Hum Genome Var. 2025 Sep 26;12:20. doi: 10.1038/s41439-025-00324-0 (PMC12474988; doi:10.1038/s41439-025-00324-0)
Supplement: Supplementary file 1 — Supplementary Information [file 41439_2025_324_MOESM1_ESM.docx]

**SUPPLEMENTARY INFORMATION**

**Juvenile/adult type galactosialidosis with a homozygous *CTSA* variant without consanguinity**

Machiko Toki, Kazushige Tsunoda, Tetsumin So, Motomichi Kosuga, Torayuki Okuyama,

Masashi Miharu, Tomonobu Hasegawa, Kazuki Yamazawa

**Supplementary Methods**

This study was approved from the local institutional review board, and written informed consent was obtained from the patient. Genomic DNA was extracted from peripheral blood leukocytes using QIAamp DNA Mini kit (Qiagen, Hilden, Germany). For genetic testing of GS, DNA libraries were prepared using the NEXTFLEX Rapid XP DNA-Seq Kit (Perkin Elmer, Austin, TX, USA), enriched with the Twist Custom Panel (Twist Bioscience, San Francisco, CA, USA), and sequenced on the Illumina NextSeq 2000 platform (Illumina, San Diego, CA, USA) in 75 bp paired-end mode.

To further investigate potential genetic contributors, exome sequencing (ES) was additionally performed. Libraries enriched with the SureSelect Human All Exon V6 kit (Agilent Technologies, Santa Clara, CA, USA) were sequenced on the NovaSeq 6000 platform (Illumina) in 150 bp paired-end mode. Data analysis was conducted as previously described^1,2^. To exclude the possibility of unrecognized distant relatedness, homozygosity mapping was performed using ES data analyzed with PLINK^3^ and AutoMap^4^ under the default settings. Furthermore, chromosomal microarray analysis was conducted using the GenetiSure Dx Postnatal Assay (Agilent Technologies) according to the manufacturer’s default parameters.

The allele frequency of the detected variant in various populations was obtained from public genome databases as follows: gnomAD v4.1.0 (https://gnomad.broadinstitute.org) for global and East Asian populations^5^, ToMMo 61KJPN (https://jmorp.megabank.tohoku.ac.jp) for the Japanese population^6^, the ChinaMAP (http://www.mbiobank.com) for the Chinese population^7^, Korean Variant Archive 2 (KOVA 2, https://www.kobic.re.kr/kova) for the Korean population^8^, Thai Reference Exome Database (T-REx, https://trex.nbt.or.th) for the Thai population^9^, and the 1000 Vietnamese Genome Project (VN1K) (https://genome.vinbigdata.org/) for the Vietnamese population. We also searched disease databases such as ​ClinVar (https://www.ncbi.nlm.nih.gov/clinvar/) and Human Gene Mutation Database (HGMD^®^, https://www.hgmd.cf.ac.uk/ac/index.php). For *in silico* analyses, we utilized two prediction algorithms: Combined Annotation Dependent Depletion^10^ (CADD, https://cadd.gs.washington.edu/) and SpliceAI^11^ (https://spliceailookup.broadinstitute.org/).

**Supplementary Fig. 1**

Homozygosity mapping using AutoMap demonstrated a total of 22.07 Mb of ROHs across the autosomes, including 1.95 Mb ROH encompassing the *CTSA* gene locus on chromosome 20.

| **Supplementary Table 1** Enzymatic activity testing in peripheral blood lymphocytes | | | | |
| --- | --- | --- | --- | --- |
| Enzyme | Our patient | Positive control | Normal control | Reference (nmol/h/mg) |
| β-Galactosidase | 11 | 12 | 125 | 99 ± 22 |
| Sialidase | 0.03 | 0.11 | 0.22 | Not available |

| **Supplementary Table 2** Database search and *in silico* analysis of the *CTSA* c.692+3A>G variant | | | |  |
| --- | --- | --- | --- | --- |
| Population database | | | | |
|  | Database | Population | Allele frequency | Allele count or Number of samples |
|  | gnomAD v4.1.0 | Global | 4.97 × 10^-6^ | 8/1,610,666 |
|  | gnomAD v4.1.0 | East Asian | 1.78 × 10^-4^ | 8/44,850 |
|  | ToMMo 61KJPN | Japanese | 1.48 × 10^-4^ | 18/121,704 |
|  | ChinaMAP | Chinese | Absent | 10,588 |
|  | KOVA.v2 | South Korean | Absent | 5,305 |
|  | T-Rex | Thai | Absent | 1,092 |
|  | VN1K | Vietnamese | Absent | 1,000 |
| Disease database | | | | |
|  | ClinVar | Pathogenic |  |  |
|  | HGMD^®^ | Disease causing mutation |  |  |
| *in silico* analysis | | | | |
|  | CADD PHRED | 20.5 |  |  |
|  | SpliceAI | 0.55 | Acceptor Loss, -94 bp |  |
|  |  | 0.61 | Donor Loss, -3 bp |  |
| All data were obtained by accessing each database in June 2025. | | | | |

**References**

1. Yamazawa K, Inoue T, Sakemi Y, Nakashima T, Yamashita H, Khono K *et al.* Loss of imprinting of the human-specific imprinted gene ZNF597 causes prenatal growth retardation and dysmorphic features: implications for phenotypic overlap with Silver-Russell syndrome. *J Med Genet* **58**, 427-432 (2021).

2. Nakashima M, Shiroshima T, Fukaya M, Sugawara T, Sakagami H & Yamazawa K. C-terminal truncations in IQSEC2: implications for synaptic localization, guanine nucleotide exchange factor activity, and neurological manifestations. *J Hum Genet* **69**, 119-123 (2024).

3. Purcell S, Neale B, Todd-Brown K, Thomas L, Ferreira MA, Bender D *et al.* PLINK: a tool set for whole-genome association and population-based linkage analyses. *Am J Hum Genet* **81**, 559-575 (2007).

4. Quinodoz M, Peter VG, Bedoni N, Royer Bertrand B, Cisarova K, Salmaninejad A *et al.* AutoMap is a high performance homozygosity mapping tool using next-generation sequencing data. *Nat Commun* **12**, 518 (2021).

5. Chen S, Francioli LC, Goodrich JK, Collins RL, Kanai M, Wang Q *et al.* A genomic mutational constraint map using variation in 76,156 human genomes. *Nature* **625**, 92-100 (2024).

6. Tadaka S, Kawashima J, Hishinuma E, Saito S, Okamura Y, Otsuki A *et al.* jMorp: Japanese Multi-Omics Reference Panel update report 2023. *Nucleic Acids Res* **52**, D622-D632 (2024).

7. Cao Y, Li L, Xu M, Feng Z, Sun X, Lu J *et al.* The ChinaMAP analytics of deep whole genome sequences in 10,588 individuals. *Cell Res* **30**, 717-731 (2020).

8. Lee J, Lee J, Jeon S, Lee J, Jang I, Yang JO *et al.* A database of 5305 healthy Korean individuals reveals genetic and clinical implications for an East Asian population. *Exp Mol Med* **54**, 1862-1871 (2022).

9. Shotelersuk V, Wichadakul D, Ngamphiw C, Srichomthong C, Phokaew C, Wilantho A *et al.* The Thai reference exome (T-REx) variant database. *Clin Genet* **100**, 703-712 (2021).

10. Rentzsch P, Witten D, Cooper GM, Shendure J & Kircher M. CADD: predicting the deleteriousness of variants throughout the human genome. *Nucleic Acids Res* **47**, D886-d894 (2019).

11. Jaganathan K, Kyriazopoulou Panagiotopoulou S, McRae JF, Darbandi SF, Knowles D, Li YI *et al.* Predicting Splicing from Primary Sequence with Deep Learning. *Cell* **176**, 535-548.e24 (2019).
